# Calcium channel blocker in patients with chronic kidney disease

**DOI:** 10.1007/s10157-021-02153-1

**Published:** 2021-11-08

**Authors:** Shoko Ohno, Akira Ishii, Motoko Yanagita, Hideki Yokoi

**Affiliations:** 1 Department of Nephrology, Graduate School of Medicine, Kyoto University, 54 Shogoin Kawahara-cho, Sakyo-ku, Kyoto, 606-8507 Japan; 2 Institute for the Advanced Study of Human Biology (ASHBi), Kyoto University, Kyoto, Japan

**Keywords:** Calcium channel blocker, Hypertension, Chronic kidney disease

## Abstract

**Background:**

Chronic kidney disease (CKD) is involved in a progressive deterioration in renal function over the years and is now a global public health problem. Currently, reducing the number of patients progressing to end-stage renal failure is urgently necessary. Hypertension and CKD interact with each other, and good control of blood pressure (BP) can improve CKD patients’ prognosis. With the current global trend for more strict BP control, the importance of BP management and the need for medication to achieve this strict goal are increasing. Calcium channel blockers (CCBs), which target voltage-dependent calcium channels, are frequently used in combination with renin–angiotensin–aldosterone system inhibitors for CKD patients because of their strong BP-lowering properties and relatively few adverse side effects. Calcium channels have several subtypes, including L, N, T, P/Q, and R, and three types of CCBs, L-type CCBs, L-/T-type CCBs, and L-/N-type CCBs, that are available. Nowadays, the new functions and effects of the CCBs are being elucidated.

**Conclusion:**

We should use different types of CCBs properly depending on their pharmacological effects, such as the strength of antihypertensive effects and the organ protection effects, taking into account the pathophysiology of the patients. In this article, the role and the use of CCBs in CKD patients are reviewed.

## Introduction

Chronic kidney disease (CKD) causes a progressive deterioration in renal function over a long period and has become a worldwide public health problem [1]. More than 13 million people have been estimated to suffer from various stages of CKD in Japan, with more than 340,000 people undergoing dialysis treatment. The growing number of dialysis patients put a strain on the healthcare economy. Currently, CKD is an emerging epidemic and reducing the number of patients progressing to end-stage of renal disease is urgently needed.

Hypertension is a common cause of CKD, and since CKD leads to hypertension, hypertension and CKD strongly influence each other. Hypertension is present in approximately 80% of patients with CKD [2]. Hypertension in CKD patients has several causes, and the first is impaired sodium excretion, which causes an increase of extracellular fluid volume. The second is activation of the renin–angiotensin–aldosterone system (RAAS), the third is stimulation of the sympathetic nerve system and the resulting direct persistence of vasoconstriction, and the fourth is calcification of arterial vessels. There are numerous other factors in CKD patients influencing hypertension.

Blood pressure (BP) control is effective in preventing complications including cardiovascular disease (CVD). Particularly, CVD is a major cause of morbidity and mortality in patients with CKD. Therefore, one of the aims of BP control for CKD patients is to prevent CVD. Furthermore, lowering BP can slow the rate of renal disease progression. The GFR reduction rate slows down by a good BP control [3, 4]. Therefore, effective management of hypertension is essential for the management of CKD. In addition, the concept of diabetic kidney disease (DKD) has been proposed, and the involvement of nephrosclerosis as a background factor for DKD is attracting attention [5]. Blood pressure control is also important in diabetic patients for protecting renal function.

Calcium channel blockers (CCBs), which target voltage-dependent calcium channels, are frequently used in combination with RAAS inhibitors for CKD patients because of their strong BP-lowering properties and relatively few adverse side effects [6, 7]. In Japan, CCBs are commonly used as the first-line medication for hypertension, along with RAAS inhibitors and diuretics. There are several subtypes of calcium channels, such as L, N, T, P/Q, and R [8, 9]. CCBs include L-type CCBs, L-/T-type CCBs, and L-/N-type CCBs, which can be used in hypertensive patients depending on their pharmacological function [10].

In this article, we reviewed the localizations and roles of calcium channels and the effects of CCBs on CKD patients.

## Guidelines for BP treatment for CKD patients

Various societies in different countries have developed their own guidelines related to hypertension. They have not established common BP targets and optimal BP goals for CKD patients. The guideline of Kidney Disease Improving Global Outcomes (KDIGO) indicates that the target BP for hypertensive and CKD patients should be < 120 mmHg systolic, when tolerated [11]. A Report of the International Society of Hypertension on 2020 Global Hypertension Practice Guidelines recommends a strict BP goal of 130/80 mmHg as a diagnosis of hypertension in adults with CKD [12]. Recent trends call for strict BP control. This may be due to the influence of several trials such as SPRINT (Systolic Pressure Intensive Trial) [13]. The same trend can be seen in the Japanese guidelines.

The Japanese Society of Nephrology published the evidence-based clinical practice guideline for CKD in 2018. The BP target for CKD patients is different according to the presence or absence of diabetes. The BP target is < 130 mmHg and < 80 mmHg for systolic and diastolic, respectively if diabetes is complicated. In contrast, if there is no diabetes nor albuminuria, BP targets are < 140 mmHg and < 90 mmHg for systolic and diastolic, respectively. If there is no diabetes but an albumin excretion rate of ≥ 30 mg/24 h, the BP targets are < 130 mmHg and < 80 mmHg for systolic and diastolic, respectively. For elderly people over 75 years of age, the recommended BP targets are < 150 mmHg and < 90 mmHg for systolic and diastolic, respectively.

The Japanese Society of Hypertension published the guideline for the management of hypertension in 2019. BP targets are < 130 mmHg and < 80 mmHg for systolic and diastolic, respectively, in CKD patients with proteinuria. Over 75 years old CKD patients without proteinuria, BP targets are < 140 mmHg and < 90 mmHg for systolic and diastolic, respectively [14].

## Pharmacological management of hypertension for CKD patients

Pharmacological management is important to treat hypertension, reach the target BP goals, and manage BP more effectively. The medications can be effective in reducing urinary protein, slowing the renal function aggravation, and preventing various events including CVD. More than one drug may be required in patients with treatment-resistant hypertension [7, 15], and this is especially true in patients with CKD. The selection of an appropriate antihypertensive regimen for CKD patients is discussed in this review.

RAAS inhibitors ACEI/ARB should be the first-line treatment to manage hypertension for CKD patients with proteinuria/albuminuria according to the Japanese Society of Hypertension Guidelines [14]. Several meta-analyses and RCTs have shown that RAAS inhibitors slow the progression of CKD and reduce mortality [16]. Furthermore, RAAS inhibitors show a more renal protective effect by reducing proteinuria. However, RAAS inhibitors should be administered with caution in patients with CKD stages 4 and 5, as they may cause renal function deterioration and hyperkalemia. CCBs and thiazide diuretics are also recommended for CKD patients.

Susantitaphong et al*.* reported that dual RAAS blockade therapy reduced both BP and albuminuria [17]. However, dual therapy often increases the risk of hyperkalemia and acute kidney injury [18, 19]. The combination therapy of ACEi and ARB is not recommended in preventing renal function deterioration in current guidelines.

Mineralocorticoid receptor antagonists (MRAs) in addition to ACEi or ARB reduce both BP and albuminuria in CKD patients with albuminuria. More recently, one of the MRAs, finerenone, was reported to improve renal outcomes—renal failure, eGFR reduction of more than 40%, and death from renal causes—of diabetic nephropathy patients with albuminuria, in addition to reducing albuminuria [20]. Conversely, an increased risk of hyperkalemia exists when MRAs are prescribed in addition to ACEi and ARBs for CKD patients; MRAs should also be carefully administered in CKD patients with caution for hyperkalemia.

## CCB in the pharmacological management of hypertension

KDIGO’s 2021 guidelines recommend CCBs along with RAAS inhibitors and thiazide diuretics for cardiovascular disease prevention in primary hypertension [11]. When starting antihypertensive therapy, it is recommended to begin with 1 or more drugs among RAAS inhibitors, CCB, and thiazide-like diuretic. In the report of International Society of hypertension on 2020 Global hypertension practice Guideline, CCBs are recommended as a first-line drugs in hypertensive patients with a history of stroke, as are RAAS inhibitors and diuretics [12]. About hypertension patients with CKD, RAS-inhibitors are recommended as a first-line drug because they reduce albuminuria in addition to BP control, and CCBs and diuretics are mentioned as agents that can be added further. The guideline also mentioned that during pregnancy, dihydropyridine calcium channel blockers (DHP-CCBs) (nifedipine (not in capsule form), nicardipine) are the first choice, along with methyldopa and beta-blockers (labetalol). Moreover, in breastfeeding patients, long acting CCBs are preferred. Thus, in clinical practice, CCB are frequently used and have a wide range of indications.

For CKD patients, the evidence-based clinical practice guideline for CKD of the Japanese Society of Nephrology states the following. RAS inhibitors are recommended for CKD patients complicated with DM. RAS inhibitor is also first choice, if there is no diabetes but proteinuria of 0.15 g/gCr or more. On the other hand, RAS inhibitors, CCB, and thiazide diuretics are recommended for patients without DM with proteinuria of less than 0.15 g/gCr.

## Calcium channels: physiological roles and pharmacological modification

### Classification of calcium channels

**Fig. 1:**
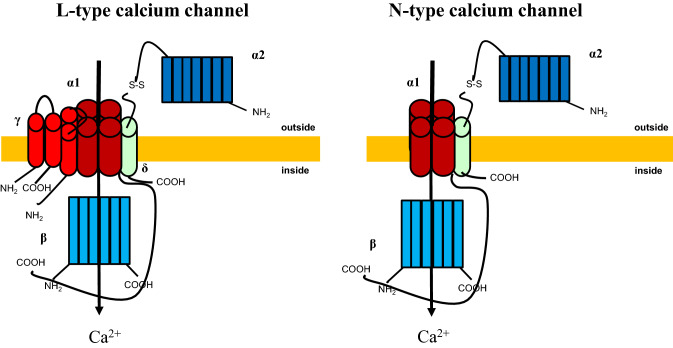
Structure of calcium channel. The voltage-dependent calcium channels are localized in the plasma membrane and are essential for the release of neurotransmitters and hormones. These channels are classified into L-, P/Q-, N-, R-, and T-type subtypes based on their pharmacological and electrophysiological properties. Molecular biological analysis has shown that calcium channels are composed of *α*1, *α*2/*δ*, *β*, and *γ* subunits, among which *α*1 subunits are most important for defining channel properties. The N-type and L-type structures are shown. The *γ* subunit is deficient in N-type calcium channels

**Fig. 2:**
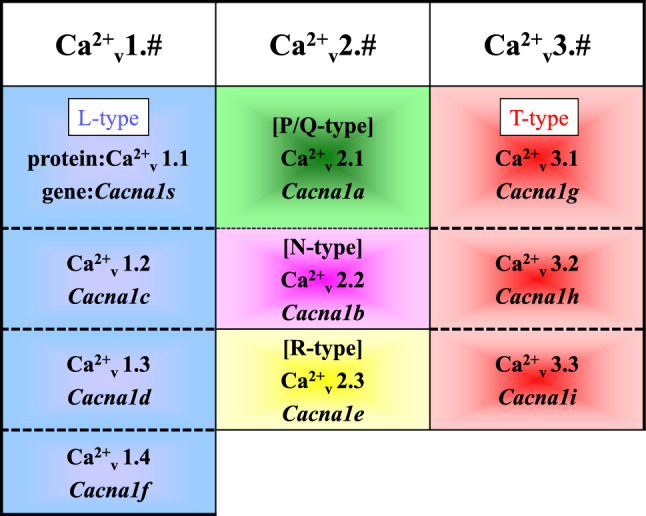
Classification of calcium channel. The calcium channels are classified into L-, P/Q-, N-, R-, and T-type subtypes based on their pharmacological and electrophysiological properties. The *α*1 subunit genes have been cloned and classified into the following three subfamilies based on their sequence similarity: Ca_*v*_1.x, Ca_*v*_2.x, and Ca_*v*_3.x. Ten calcium channel *α*1 gene names are described in italic

The voltage-dependent calcium channels are localized in the plasma membrane and are essential for the release of neurotransmitters and hormones [21]. These channels are classified into L-, P/Q-, N-, R-, and T-type subtypes based on their pharmacological and electrophysiological properties. Molecular biological analysis has shown that calcium channels are composed of *α*1,* α*2/*δ*, *β*, and *γ* subunits [22], among which α1 subunits are the most important in defining channel properties (Fig. 1). The α1 subunits are encoded by *CACNA1* gene family consisting of 10 genes. These α1 subunit genes were cloned and classified into the following three subfamilies based on their sequence similarity: Ca_*v*_1.x, Ca_*v*_2.x, and Ca_*v*_3.x (Fig. 2).

### Localization of calcium channels in the kidney

**Fig. 3:**
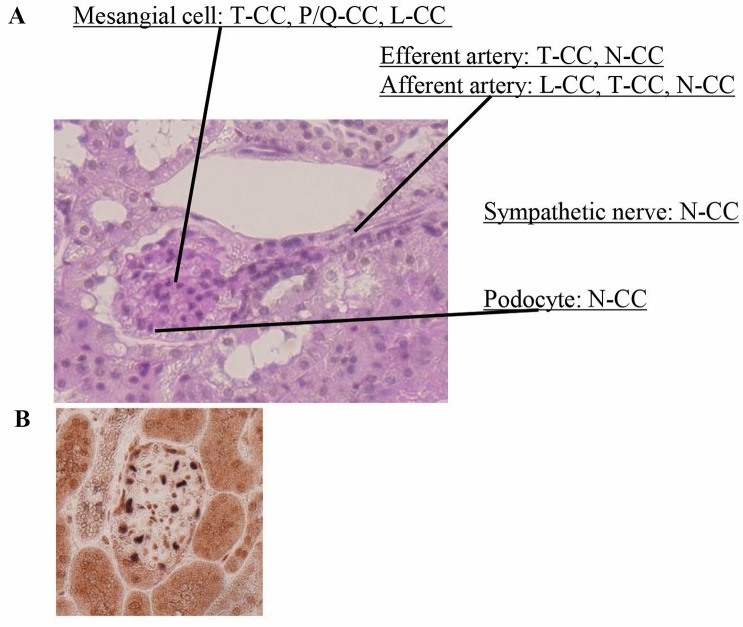
Localization of calcium channels in glomeruli. **A** L-type, P/Q-type and T-type are reported to localize in mesangial cells. T-type is expressed on podocytes and is also involved in the sympathetic nerve. Regarding afferent and efferent arterioles, both T-type and N-type are expressed, but L-type is reported only in afferent arterioles. **B** Double immunostaining for Ca_*v*_2.2 (brown) and WT1 (blue) shows double positive cells in a glomerulus. This figure is modified from the article [31]. *CC* calcium channel

In the kidney, several calcium channels are expressed, including Ca^2+^_*V*_1.2 (*α*1C), Ca^2+^_*V*_1.3 (*α*1D), Ca^2+^_*V*_2.1 (*α*1A), Ca^2+^_*V*_2.2 (*α*1B), Ca^2+^_*V*_3.1(*α*1G), and Ca^2+^_*V*_3.2 (*α*1H). They are classified according to function into L-type (Ca^2+^_*V*_1.2, Ca^2+^_*V*_1.3), P/Q-type (Ca^2+^_*V*_2.1), N-Type (Ca^2+^_*V*_2.2), and T-type (Ca^2+^_*V*_3.1, Ca^2+^_*V*_3.2) Ca^2+^ channels [8, 23, 24]. Figure 3 and Table 1 shows the localization of calcium channels in the kidney.

**Table 1:** Localization of calcium channel in the kidney of rats and mice

	Glomerulus	Afferent/efferent arteriole	Distal tubules	Collecting duct
L	Mesangial cell Hansen et al. [39]	Hansen et al. [39] Hayashi et al. [23]	Zhao et al. [25]	Zhao et al. [25]
N	Podocyte Fan et al. [30] Ohno et al. [31]		Fan et al. [30] Ohno et al. [31]	
T	Mesangial cell Hansen et al. [39]	Hansen et al. [39] Poulsen et al. [27]	Andreasen et al. [26]	Andreasen et al. [26]
P/Q	Mesangial cell Hansen et al. [40]	Hansen et al. [40]		

First, the following basic researches have been reported on rats and mice. L-type calcium channel, Ca^2+^_*V*_1.2, is expressed in cells of the distal tubules as well as in the outer and inner medullary collecting ducts of a rat kidney by immunohistochemistry [25]. Andreasen et al*.* examined the nephron localization of Ca^2+^_*V*_3.1, T-type calcium channel in rats, and found it to be expressed in the distal convoluted tubules and collecting duct [26]. On the other hand, Poulsen et al*.* reported that T-type calcium channels, Ca^2+^_*V*_3.1 and Ca^2+^_*V*_3.2, are expressed in the efferent arterioles of a mouse kidney [27]. Few reports on the localization of calcium channels have explicitly mentioned humans. Hansen et al*.* reported that L-type (Ca^2+^_*V*_1.2), P/Q-type (Ca^2+^_*V*_2.1), and T-type (Ca^2+^_*V*_3.1, Ca^2+^_*V*_3.2) calcium channels are expressed in the human renal artery and dissected intrarenal blood vessels by PCR analysis [28]. Hansen et al*.* also reported that Ca^2+^_*V*_2.1, a P/Q-type calcium channel, contributes functionally to renal blood vessel contraction in humans [29].

Fan et al*.* demonstrated N-type (Ca^2+^_*v*_2.2) calcium channels’ immunoreactivity in rat renal vascular walls, possibly nerves in the adventitia, and in the distal tubules and podocytes [30]. They also reported that the N-type calcium channel in cultured podocytes was involved in producing reactive oxygen species by angiotensin-II [30]. Previously, we reported that Ca^2+^_*v*_2.2 was localized in the glomerulus including podocytes and in distal tubular cells of mouse by immunohistochemical study [31].

### Function of calcium channels on renal microvasculature

The renal microvasculature comprises two major categories: the glomerular capillary and peritubular capillary (PTC). When the arterial blood flow enters the kidney, it gradually shifts to the smaller arteries and enters the glomerulus capillaries through the afferent arterioles. Post-glomerular blood flow enters the peritubular capillaries (PTCs) that feed the tubules.

L-type calcium channel blockade does not reduce the intraglomerular pressure because the L-type calcium channels are present only in the afferent arterioles. Their blockade mainly dilates the afferent arterioles in a rat kidney [32]. On the other hand, T-type calcium channels are present in both the afferent and efferent arterioles of the rat kidney. Therefore, T-type calcium channel blockade dilates both arterioles and reduces the glomerular capillary pressure, drawing attention to the role of T-type channels in renal protection [33, 34]. However, Thuesen et al*.* conducted experiments using T-type CC knockout mice and found that deficiency of Ca_*V*_3.1 increased renal blood flow (RBF) and deficiency of Ca_*V*_3.2 increased glomerular filtration rate (GFR), suggesting that Ca_*V*_3.1 is involved in afferent tone and Ca_*V*_3.2 influences efferent dilatation [35]. N-type calcium channels are present at the synaptic nerve endings of both the afferent and efferent arterioles [36]. Cilnidipine, an N/L-type calcium channel blocker, ameliorates glomerular hypertension by dilating both the afferent and efferent arterioles in the canine kidney [23]. Since L-type CCBs act on afferent arterioles, cilnidipine improves glomerular hypertension by blocking N-type calcium channels. The reduction of glomerular pressure is one of the important factors in reducing proteinuria in hypertensive patients [37]. To improve glomerular hypertension, controlling the arteriolar resistance, especially in the efferent arterioles, is highly effective [38].

### Function of calcium channels on glomerulus

Although there are several studies referred to the localization of calcium channels in the blood vessels as above, there exist few reports referred to the localization in glomeruli. Three types of calcium channels, L-type [39], P/Q-type [40], and T-type [39], have been reported to localize in the mesangial cells. Previous reports showed that glomerular podocytes express N-type calcium channels [30, 41].

Konda et al*.* reported that cilnidipine, an N/L-type CCB, reduces BP and improves glomerular sclerosis in Dahl salt-sensitive rats [42]. Fan et al*.* also reported cilnidipine suppressed more proteinuria level than amlodipine by inhibiting N-type calcium channel-dependent podocyte injury in spontaneously hypertensive rats/ND mcr-cp (SHR/ND) [30]. Cilnidipine significantly prevented the podocyte injury assessed by desmin staining and restored the glomerular podocin and nephrin expression compared with amlodipine in SHR/ND rats.

**Fig. 4:**
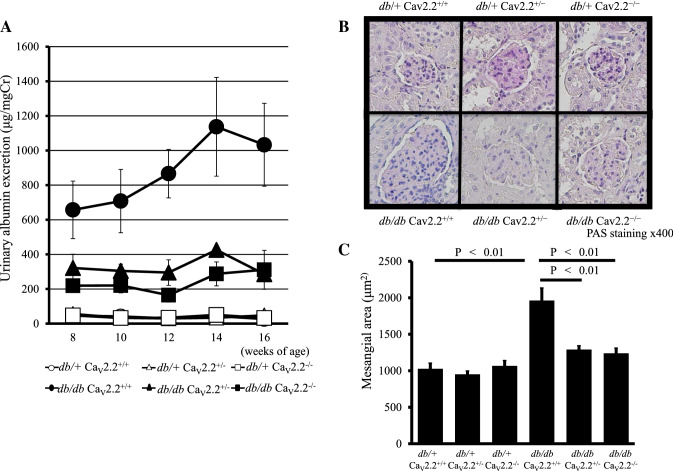
Alleviation of mesangial expansion and urinary albumin excretion in diabetic Ca_*v*_2.2^−/−^ mice. **A** Time course of urinary albumin excretion per milligram creatinine of experimental mice. Urinary albumin excretion of both *db/db* Ca_*v*_2.2^−/−^ and *db/db* Ca_v_2.2^+/–^ mice was lower than that of *db/db* Ca_*v*_2.2^+*/*+^ mice. *db/* + Ca_*v*_2.2^+*/*+^ mice (white circles), *db/* + Ca_*v*_2.2^+/–^ mice (white triangles), *db/* + Ca_*v*_2.2^−/−^ mice (white squares), *db/db* Ca_*v*_2.2^+*/*+^ mice (black circles), *db/db* Ca_*v*_2.2^±^ mice (black triangles), and *db/db* Ca_*v*_2.2^−/−^ mice (black squares). **B** A glomerulus of mice at 16 weeks of age in each mouse. **C** Mesangial area was increased in diabetic Ca_*v*_2.2^+/+^ mice and was suppressed in diabetic Ca_*v*_2.2^−/−^ mice. This figure is modified from the article [31]

We employed mice lacking the N-type calcium channel *α*1 subunit gene (Ca_*v*_2.2^−/−^) to generate *db/db* (diabetic) and Ca_*v*_2.2^−/−^ double mutant mice [31]. In our study, albuminuria and glomerular histological changes were significantly alleviated in diabetic Ca_*v*_2.2^−/−^ mice (Fig. 4). In cultured podocytes, depolarization-dependent calcium responses were decreased by *ω*-conotoxin, a Ca_*v*_2.2-specific inhibitor. Furthermore, the reduction of nephrin by transforming growth factor-*β* (TGF-*β*) in podocytes was abolished with *ω*-conotoxin. These results suggest that Ca_*v*_2.2 inhibition exerts renoprotective effects against the progression of diabetic nephropathy, partly by protecting podocytes.

CCBs are widely-used antihypertensive drugs and a large body of human evidence supports their pleiotropic effect. L-type CCB is the most widely prescribed drug worldwide and has the longest history. Recently, however, the renal protective effects of new types of T-type and N-type CCBs have received more attention [43, 44].

Clinical studies reported that L-/T-type CCB (benidipine) is superior to treatment with L-type CCB (amlodipine) in decreasing urinary albumin excretion and plasma aldosterone level [45–47]. In the JATOS trial, a large-scale clinical trial evaluating the optimal target BP in elderly hypertensive patients, the renal subanalysis showed that efonidipine, T-/L-type CCB, improved eGFR in patients with proteinuria [48].

N-/L-type CCBs have also been reported to have renal protective effects. Konoshita et al*.* reported that cilnidipine, an N-/L-type CCB, leads to less activation of RAAS in hypertensive patients than does amlodipine, and that UAE with cilnidipine administration was also significantly lower than that with amlodipine [49]. The CARTER study reported that cilnidipine reduced proteinuria in patients with CKD who were already treated with RAAS inhibitors [50].

### Function of calcium channels on renal tubules

There are several reports referred to the localization in the tubules. Brunette et al*.* reported that L-, P/Q- and T-type calcium channels exist in the luminal side of the distal tubules in rabbits, because their channel antagonist, including diltiazem, *ω*-conotoxin, and mibefradil, decreased Ca^2+^ transport in the absence of sodium [51]. Andreasen et al*.* showed the nephron localization of Ca^2+^_*V*_3.1, T-type calcium channels, in the inner medullary collecting ducts, distal collecting ducts and collecting tubules, especially in the apical sites [26]. Sugano et al*.* investigated the effect of the stereoselective T-type calcium channel blocker *R*(-)-efonidipine and CKD progression in spontaneously hypertensive rats that had undergone subtotal nephrectomy [52]. They showed that T-type calcium channel blockade has renal protective actions that depend not on hemodynamic changes and on the inhibition of Rho-kinase activity, tubulointerstitial fibrosis, and epithelial-mesenchymal transitions. Baylis et al*.* reported that T-/L-type CCBs, mibefradil, resulted in superior nephroprotection including amelioration of proteinuria and glomerular injury, to traditional L-type CCBs, amlodipine, in deoxycorticosterone acetate (DOCA)-salt rats which are the models of high glomerular BP and rapidly developing kidney damage [53]. In contrast, L-type CCBs, amlodipine, failed to improve renal injury. They suggest that amelioration of tubulointerstitial injury may contribute to the nonhemodynamic effect of T-type CCBs. However, the relationship between calcium channels and renal tubules is not fully investigated. Further studies are needed to address these questions.

#### Calcium channels in the sympathetic nerve system

Ino et al*.* reported that N-type calcium channel α1 subunit knockout (Ca_*v*_2.2^−/−^) mice that lack the cytosolic portion of the N-type calcium channel are viable and have an almost normal behavior but show a very low sympathetic nerve activity in the atria [54]. Previously, we reported that diabetic Ca_*v*_2.2^−/−^ mice showed low SBP with a marked reduction in urinary catecholamine levels [31]. Yamada et al*.* showed that Ca_*v*_2.2^−/−^ mice exhibited lower SBP than control mice because of vasodilatation, reduced heart contractile activity, and inhibited sympathetic nerve activity [55]. Recently, it is shown that N-type Ca^2+^ channel is upregulated in the interstitial nerve fibers of obstructed fibrotic kidneys of mouse and ablation of N-type Ca^2+^ channel significantly attenuated the fibrotic changes of the kidneys after UUO partly by the reduction of renal sympathetic nerve activation [56]. The P/Q-type calcium channels are also associated with neurons but may not be with sympathetic nerves. Mutations in P/Q-type calcium channel are involved in neurological disorders, including epilepsy and familial hemiplegic migraine [56].

#### The relationship between RAAS system and CCBs

In L/T type and L/N type CCBs, they are expected to have renoprotective by acting on efferent arteriole dilatation. However, they are reported to be no more effective than RAAS inhibitors. Li et al. reported in their systematic review and meta-analysis that anti-proteinuric effects of T-type CCBs are stronger thatn L-type CCBs, and do not differ from these of RAS inhibitors [46]. RAAS inhibitors and CCBs are often used in combination. Some reports showed that RAAS inhibitors are effective in reducing edema, a side effect of CCBs, by decreasing capillary hypertension and transcapillary fluid exudation [57, 58]. Jamerson et al*.* reported that the combination therapy of CCBs and RAAS inhibitors reduces the risk of cardiovascular events [59].

Thuesen et al. reported that deficiency of T-type calcium channel, Ca_*v*_3.1 or Ca_*v*_3.2, do not alter baseline blood pressure levels and Ang II-induced hypertension [35]. However, Ca_v_3.1, but not Ca_v_3.2, contributes to aldosterone secretion in mice infused Ang II [35]. Imagawa et al*.* reported that efonidipine, L/T type CCB, significantly reduces aldosterone synthesis and secretion [60].

N-type calcium channel is expressed in the sympathetic nerve terminals and regulates catecholamines’ release [61]. Therefore, N/L-type CCB reduces plasma catecholamine secretion rate through inhibiting N-type calcium channel [62], and can be less active in RAAS than L-type CCB in animal models [49, 63, 64].

There are still many unanswered questions about CCBs and RAAS inhibitors, and further studies on the effects of combination therapy are necessary.

## Conclusion

Hypertension and CKD interact with each other, and good BP control can improve CKD patients’ prognosis. Worldwide, BP management is becoming increasingly important with the current trend for more stringent BP control. To achieve this strict goal, the need for medication is increasing. CCBs play a major role in antihypertensive medication. Understanding the types of CCBs, the strength of their antihypertensive effects, the difference in organ protection effects, and using them properly according to the patients and their pathophysiology are necessary. N-/L-type and T-/L-type CCBs can have additional organ-protecting effects in addition to the reliable antihypertensive effect of conventional L-type CCBs. It requires further investigations to clarify the mechanisms of organ-protecting effects of CCBs. Establishing evidence for the effectiveness of various CCBs and establishing guidelines for more effective CCB administration are also necessary.
